# Supplementary material for: Sprayable RNAi for silencing of important genes to manage red palm weevil, Rhynchophorus ferrugineus (Coleoptera: Curculionidae)
Source: PLoS One. 2024 Oct 24;19(10):e0308613. doi: 10.1371/journal.pone.0308613 (PMC11501036; doi:10.1371/journal.pone.0308613)
Supplement: Attachment — Submitted filename: PONE-D-24-01103_R1.pdf [file pone.0308613.s002.pdf]

PLOS ONE

Sprayable RNAi for silencing of important genes to manage red palm weevil, Rhynchophorus ferrugineus (Coleoptera: Curculionidae)

--Manuscript Draft--

**Manuscript Number:**	PONE-D-24-01103
**Article Type:**	Research Article
**Full Title:**	Sprayable RNAi for silencing of important genes to manage red palm weevil, Rhynchophorus ferrugineus (Coleoptera: Curculionidae)
**Short Title:**	Gene silencing for RPW management
**Corresponding Author:**	Muhammad Nadir Naqqash, PhD<br>MNS-University of Agriculture: Muhammad Nawaz Shareef University of Agriculture<br>Nigde, TURKEY
**Keywords:**	Date palm; dsRNAs; Management; qRT-PCR; Rhynchophorus ferrugineus; RNAi
**Abstract:**	Red palm weevil (RPW) is the most devastating insect-pest of palm trees worldwide. Synthetic insecticides are the most preferred tool for the management of RPW. Alternatively, RNA interference (RNAi) mediated silencing of crucial genes provides reasonable control of insect pests. Recently we have targeted four important genes; ecdysone receptor (EcR), serine carboxypeptidase (SCP), actin and chitin-binding peritrophin (CBP) in 3rd and 5th instar larvae RPW. The results from 20 days trial showed that the survival rate of 3rd instar larvae fed on SCP and actin dsRNAs had the lowest survival (12-68%) days of incubation. While, in the 5th instar larvae, the lowest survival rate (24%) was recorded for SCP after 20 days of incubation. Similarly, the weight of the 3rd and 5th instar larvae treated with SCP and actin was significantly reduced to 2.30-2.36 g and 4.64-4.78 g after 6 days of dsRNA exposure. The larval duration was also decreased significantly in the larvae treated with all the dsRNA treatments. The qRT-PCR results confirmed a significant suppression of the targeted genes as 90-97% and 85-93% in the third and fifth instar larvae, respectively. The results in this study suggest that the SCP and the actin genes can be promising targets to mediate RNAi-based control of RPW.
**Order of Authors:**	Muhammad Nadir Naqqash, PhD
	Muhammad Naeem Sattar
	Adel A. Rezk
	Khalid Mehmood
	Allah Bakhsh
	Hamadttu Elshafie
	Jameel M. Al-Khayri
**Additional Information:**	
**Question**	**Response**
**Financial Disclosure**<br><br>Enter a financial disclosure statement that describes the sources of funding for the work included in this submission. Review the submission guidelines for detailed requirements. View published research articles from *PLOS ONE* for specific examples.	Yes

Powered by Editorial Manager® and ProduXion Manager® from Aries Systems Corporation

This statement is required for submission and **will appear in the published article** if the submission is accepted. Please make sure it is accurate.<br><br>**Funded studies**<br>Enter a statement with the following details:<br>• Initials of the authors who received each award<br>• Grant numbers awarded to each author<br>• The full name of each funder<br>• URL of each funder website<br>• Did the sponsors or funders play any role in the study design, data collection and analysis, decision to publish, or preparation of the manuscript?<br><br>Did you receive funding for this work?	
Please add funding details.<br>as follow-up to "**Financial Disclosure**<br><br>Enter a financial disclosure statement that describes the sources of funding for the work included in this submission. Review the submission guidelines for detailed requirements. View published research articles from *PLOS ONE* for specific examples.<br><br>This statement is required for submission and **will appear in the published article** if the submission is accepted. Please make sure it is accurate.<br><br>**Funded studies**<br>Enter a statement with the following details:<br>• Initials of the authors who received each award<br>• Grant numbers awarded to each author<br>• The full name of each funder<br>• URL of each funder website<br>• Did the sponsors or funders play any role in the study design, data collection and analysis, decision to publish, or preparation of the manuscript?<br><br>Did you receive funding for this work?"	This project was financially supported King Abdulaziz City for Science and Technology (KACST), Saudi Arabia through the Project No. 11BIO1803-06

Please select the country of your main research funder (please select carefully as in some cases this is used in fee calculation).<br>as follow-up to "**Financial Disclosure**<br><br>Enter a financial disclosure statement that describes the sources of funding for the work included in this submission. Review the submission guidelines for detailed requirements. View published research articles from *PLOS ONE* for specific examples.<br><br>This statement is required for submission and **will appear in the published article** if the submission is accepted. Please make sure it is accurate.<br><br>**Funded studies**<br>Enter a statement with the following details:<br>• Initials of the authors who received each award<br>• Grant numbers awarded to each author<br>• The full name of each funder<br>• URL of each funder website<br>• Did the sponsors or funders play any role in the study design, data collection and analysis, decision to publish, or preparation of the manuscript?<br><br>Did you receive funding for this work?"	SAUDI ARABIA - SA
**Competing Interests**<br><br>Use the instructions below to enter a competing interest statement for this submission. On behalf of all authors, disclose any competing interests that could be perceived to bias this work—acknowledging all financial support and any other relevant financial or non-financial competing interests.<br><br>This statement is required for submission and **will appear in the published article** if the submission is accepted. Please make sure it is accurate and that any funding	The authors declare no conflict of interests.

sources listed in your Funding Information later in the submission form are also declared in your Financial Disclosure statement.<br><br>View published research articles from *PLOS ONE* for specific examples.<br><br>**NO authors have competing interests**<br><br>Enter: *The authors have declared that no competing interests exist.*<br><br>**Authors with competing interests**<br><br>Enter competing interest details beginning with this statement:<br><br>*I have read the journal's policy and the authors of this manuscript have the following competing interests: [insert competing interests here]*<br><br>* typeset	
**Ethics Statement**<br><br>Enter an ethics statement for this submission. This statement is required if the study involved:<br><br>• Human participants<br>• Human specimens or tissue<br>• Vertebrate animals or cephalopods<br>• Vertebrate embryos or tissues<br>• Field research<br><br>Write "N/A" if the submission does not require an ethics statement.<br><br>General guidance is provided below. Consult the submission guidelines for detailed instructions. **Make sure that all information entered here is included in the Methods section of the manuscript.**	NA

**Format for specific study types**<br><br>**Human Subject Research (involving human participants and/or tissue)**<br>• Give the name of the institutional review board or ethics committee that approved the study<br>• Include the approval number and/or a statement indicating approval of this research<br>• Indicate the form of consent obtained (written/oral) or the reason that consent was not obtained (e.g. the data were analyzed anonymously)<br><br>**Animal Research (involving vertebrate animals, embryos or tissues)**<br>• Provide the name of the Institutional Animal Care and Use Committee (IACUC) or other relevant ethics board that reviewed the study protocol, and indicate whether they approved this research or granted a formal waiver of ethical approval<br>• Include an approval number if one was obtained<br>• If the study involved *non-human primates*, add *additional details* about animal welfare and steps taken to ameliorate suffering<br>• If anesthesia, euthanasia, or any kind of animal sacrifice is part of the study, include briefly which substances and/or methods were applied<br><br>**Field Research**<br><br>Include the following details if this study involves the collection of plant, animal, or other materials from a natural setting:<br>• Field permit number<br>• Name of the institution or relevant body that granted permission	
**Data Availability**<br><br>Authors are required to make all data underlying the findings described fully available, without restriction, and from the time of publication. PLOS allows rare exceptions to address legal and ethical concerns. See the PLOS Data Policy and FAQ for detailed information.	Yes - all data are fully available without restriction

A Data Availability Statement describing where the data can be found is required at submission. Your answers to this question constitute the Data Availability Statement and **will be published in the article**, if accepted.<br><br>**Important:** Stating 'data available on request from the author' is not sufficient. If your data are only available upon request, select 'No' for the first question and explain your exceptional situation in the text box.<br><br>Do the authors confirm that all data underlying the findings described in their manuscript are fully available without restriction?	
**Describe where the data may be found in full sentences. If you are copying our sample text, replace any instances of XXX with the appropriate details.**<br><br>• If the data are **held or will be held in a public repository**, include URLs, accession numbers or DOIs. If this information will only be available after acceptance, indicate this by ticking the box below. For example: *All XXX files are available from the XXX database (accession number(s) XXX, XXX.).*<br>• If the data are all contained **within the manuscript and/or Supporting Information files**, enter the following: *All relevant data are within the manuscript and its Supporting Information files.*<br>• If neither of these applies but you are able to provide **details of access elsewhere**, with or without limitations, please do so. For example:<br><br>*Data cannot be shared publicly because of [XXX]. Data are available from the XXX Institutional Data Access / Ethics Committee (contact via XXX) for researchers who meet the criteria for access to confidential data.*<br><br>*The data underlying the results presented in the study are available from (include the name of the third party*	All relevant data are within the manuscript and its Supporting Information files

Powered by Editorial Manager® and ProduXion Manager® from Aries Systems Corporation

and contact information or URL).<br>• This text is appropriate if the data are owned by a third party and authors do not have permission to share the data.<br><br>* typeset	
Additional data availability information:	Tick here if the URLs/accession numbers/DOIs will be available only after acceptance of the manuscript for publication so that we can ensure their inclusion before publication.; Tick here if your circumstances are not covered by the questions above and you need the journal's help to make your data available.

Powered by Editorial Manager® and ProduXion Manager® from Aries Systems Corporation

Manuscript Click here to access/download;Manuscript;RPW Plos One.docx

Sprayable RNAi for silencing of important genes to manage red palm weevil, *Rhynchophorus ferrugineus* (Coleoptera: Curculionidae)

Muhammad Naeem Sattar[1,*], Muhammad Nadir Naqqash[2], Adel A. Rezk[3], Khalid Mehmood[2], Allah Bakhsh[4], Hamadttu Elshafie[5], Jameel M. Al-Khayri[3,*]

[1]Central Laboratories, King Faisal University, Al-Ahsa 31982, Saudi Arabia; mnsattar@kfu.edu.sa

[2]Institute of Plant Protection, MNS-University of Agriculture Multan, Pakistan; nadir.naqqash@mnsuam.edu.pk (MNN); sirum_mk@yahoo.com (KM)

[3]Department of Agricultural Biotechnology, College of Agriculture and Food Sciences, King Faisal University, Al-Ahsa 31982, Saudi Arabia; arazk@kfu.edu.sa (AAR); jkhayri@kfu.edu.sa (JMA)

[4]Center of Excellence in Molecular Biology, Punjab University, Lahore, Pakistan; allahbakhsh@cemb.edu.pk (AB)

[5]Date Palm Research Center of Excellence, King Faisal University, Al-Ahsa 31982, Saudi Arabia; elshafie62@yahoo.com (HE)

[*]Correspondence: MNS: mnsattar@kfu.edu.sa; Tel.: (+966-582123443)

MNN: nadir.naqqash@mnsuam.edu.pk; Tel: +923156218791)

ORCIDs:

MNS: 0000-0002-8775-4934

MNN: 0000-0001-8774-7358

AAR: 0000-0003-0259-8387

AB: 0000-0003-3561-7863

HE: 0000-0002-2055-3337

JMA: 0000-0001-9507-0201

Abstract

Red palm weevil (RPW) is the most devastating insect-pest of palm trees worldwide. Synthetic insecticides are the most preferred tool for the management of RPW. Alternatively, RNA interference (RNAi) mediated silencing of crucial genes provides reasonable control of insect pests. Recently we have targeted four important genes; ecdysone receptor (EcR), serine carboxypeptidase (SCP), actin and chitin-binding peritrophin (CBP) in 3rd and 5th instar larvae RPW. The results from 20 days trial showed that the survival rate of 3rd instar larvae fed on SCP and actin dsRNAs had the lowest survival (12-68%) ~~days of incubation~~. While, in the 5th instar larvae, the lowest survival rate (24%) was recorded for SCP after 20 days of incubation. Similarly, the weight of the 3rd and 5th instar larvae treated with SCP and actin was significantly reduced to 2.30-2.36 g and 4.64-4.78 g after 6 days of dsRNA exposure. The larval duration was also decreased significantly in the larvae treated with all the dsRNA treatments. The qRT-PCR results confirmed a significant suppression of the targeted genes as 90-97% and 85-93% in the third and fifth instar larvae, respectively. The results ~~in this study~~ suggest that the SCP and the actin genes can be promising targets to mediate RNAi-based control of RPW.

Keywords

Date palm, dsRNAs, Management, qRT-PCR, *Rhynchophorus ferrugineus*, RNAi

1. Introduction

The date palm (*Phoenix dactylifera* L.), is among the most primitive cultivated fruit trees with evidence of its cultivation dating back to 4000 B.C. It is believed to have originated in the Middle East, and from there it reached to other regions of the world, including North Africa, the Mediterranean, and South Asia. The date palm has high economic value in many countries which provides food, shelter, and revenue for millions of people [1]. Date palm is the leading fruit crop in the Kingdom of Saudi Arabia, with ~400 cultivars grown on an estimated 152 thousand hectares [2]. Saudi Arabia is the fourth top date fruit producer, with an annual production of 1.56 million tons [3]. Unfortunately, date palm trees are under the continuous threat of red palm weevil (RPW), *Rhynchophorus ferrugineus* Olivier (Coleoptera: Dryophthoridae). The RPW has been reported as an invasive insect pest that has caused higher yield losses to palm, including date palm, in many countries of the world [1–5]. Female weevils lay their eggs inside palm trees. After hatching within 3-5 days, the growing larvae feed on the terminal bud tissues and later the inner soft tissues and make tunnels up to a meter long and large cavities, causing significant damage to the plant [5–8].

Over the years, the commonly practiced approaches to control RPW, i.e., insecticides, entomopathogens or fumigants, soil/crown drenching, wound dressing, and use of pheromone traps could not provide a reasonable control [9]. The efficacy of several insecticides using different application methods, either alone or as combination is also questioned to control RPW [10–12]. Furthermore, the excessive and continuous use of synthetic insecticides may pose secondary hazards, including the resistance development human and environmental health hazards [13,14]. Insects use various detoxifying enzymes to hydrolyze insecticides as a crucial biological mechanism against several synthetic insecticides [15]. Thus, detoxifying exogenously

applied compounds' metabolism may be the primary cause of ineffective chemical insecticides [16,17]. Therefore, adopting environmental-friendly strategies, such as molecular approaches, is direly needed sustainably manage RPW. The RNA interference (RNAi) has the potential to promote the development of novel approaches toward plant protection sustainably. Numerous successful examples of gene silencing through RNAi against different groups of insects have been reported, such as in coleoptera, diptera, hemiptera, and lepidoptera [18–23].

Ecdysone receptor (EcR) plays a crucial role in insect molting, metamorphosis, and reproduction [24]. Serine carboxypeptidase (SCP) belongs to the serine protease family and is involved in body development, reproduction, juvenility and host interaction in the plant parasitic nematodes and pathogens [25,26]. Actin proteins are essential cytoskeletal proteins in cellular components and have been extensively studied as an RNAi target in many insect pests, where they cause lethal aberrations [27]. Actin is a very important protein that plays a role in many different cellular processes. It is essential for cell motility, cell division, muscle contraction, cell signaling, and endocytosis [28,29]. Chitin is a long-chain polymer of N-acetylglucosamine, and it is the key component of the insect exoskeleton. The chitin-binding peritrophins (CBPs) family of proteins bind to chitin, and they play a role in the formation, maintenance, and regulation of these extracellular structures [30,31]. Therefore, this study focused on the enzymes involved in insect development, such as EcR, SCP, actin, and CBP, for developing a safer control strategy at the molecular level through RNAi against this important insect pest. Besides, biological parameters like larval weight gain, survival and development were also studied.

2. Materials and Methods

2.1. Red Palm Weevil Population

Initially, infested date palm trees were inspected in the field and the RPW population (larva, pupa, and adult) was collected from date palm orchards in Bahawalpur (GPS location: 29.1342497089719, 71.47904164349954). The field-collected RPW individuals were carried to the insect-rearing laboratory at the Muhammad Nawaz Shareef University of Agriculture Multan (MNSUAM). Adults were housed in 1 kg plastic boxes and were fed on 10% (w/v) sugar solution via soaked cotton in the sugar solution. Eggs were separated and placed in Petri dishes (diameter: 8.00 cm; height: 2.50 cm) containing moist filter paper. After egg hatching, larvae were transferred to 1 kg plastic boxes and reared on a semisynthetic artificial diet to acquire F1 progeny for future experimentation [32]. The RPW population was maintained under controlled environmental conditions at 25°C ± 1, 12 h light-dark photoperiod and 70 ± 5% relative humidity.

2.2. Target genes selection, synthesis and cloning

Four genes, viz. EcR, SCP, actin and CBP were fully synthesized using Macrogen gene synthesis services (Macrogen Korea). All synthesized genes were cloned into pMG-Kan cloning vector (Macrogen, Korea) with selection against kanamycin. The resultant recombinant plasmids were pMG-EcR, pMG-SCP, pMG-Actin and pMG-CPB, respectively. All the recombinant plasmids were further transformed into *E. coli* cells to facilitate steady transformation.

After multiplication, the respective gene segments were further cloned separately into the RNAi cloning vector L4440 at Bgl-II and Hind-III restriction endonucleases (ThermoFisher Scientific) following Kamath and Ahringer [33]. Each construct was confirmed by PCR amplification using the respective primers for each gene (Table 1). All the RNAi constructs were further confirmed

through Sanger sequencing at Macrogen, Korea. The constructs were re-named as L4-EcR, L4-SCP, L4-Actn, and L4-CBP, respectively (Figure 1).

2.3. dsRNA synthesis in bacteria

The recombinant L4440 plasmids carrying the targeted gene fragments were transformed into chemically competent HT115 (DE3) cells following Zhu et al. [34]. The HT115 transformants (carrying the recombinant plasmid) were selected on 25 mg/mL ampicillin in an overnight culture. The diluted (100X) cultures were further grown to reach an OD600 = 0.4. Later, IPTG (10 mM) was added in the freshly prepared cultures to 1 mM concentration. Finally, the cultures were incubated at 37°C for about 5 h on regular shaking. Ultimately, the culture was given a 20 min heat shock at 80°C and stored at -20°C.

2.4. Identification of dsRNA in bacteria

Total RNA was extracted from bacteria using TRIzol reagent to analyze dsRNA production. DNase-I was used on the isolated total RNA to eliminate any remaining DNA. Concentration was determined by loading 4 g of extracted RNA onto a 1% agarose TBE gel and staining it with ethidium bromide. The pellet was resuspended in 50 L of double-distilled water. Zhu et al. [34] was followed to normalize the sample.

2.5. Biological studies to assess the impact of dsRNA

Using the diet integration feeding approach, a bioassay was conducted on RPW larvae in the 3rd and 5th instars using dsRNA [35]. The semi-synthetic diet carrying the bacterial transformants for each studied gene was prepared according to Aldawood et al. [32] and cut into 3 cm3 pellets. For the feeding bioassay, 2 µg of each dsRNA was separately incorporated into the diet, followed

by pelleting and the pellets were kept independently in cups. After 24 h starvation, the larvae were allowed to feed on dsRNA-treated pellets. The experiment lasted for six days and the diet was continuously replaced with a fresh dsRNA-treated diet every 24 h. The control group was fed only the diet treated with an empty vector. The experiment was performed with five replicates in each treatment group, and each replicate included five individual larvae. Larval mortality was recorded after 3 and 6 days. After preliminary feeding bioassay, the effects of a decreased dose of dsRNAs (1 μg) were used to study the larval survival, their development duration and final weight gain under laboratory conditions. Pupal duration and adult emergence from the pupae of treated larvae was also calculated. For this purpose, after 6 days, the larvae were transferred to an untreated diet for pupation. The experiments were conducted in a controlled environment with a temperature of 25 ± 1°C, humidity of 70 ± 5%, and a 12:12 light-dark photoperiod. The biological parameters, including mortality, survival, developmental period, and final weight gain/lost, were measured for each group. For weight gain/loss, the RPW larvae were weighed using a sensitive weighing balance (Model: ATX224; SHIMADZU) before the feeding of the dsRNA-treated diet and then weighed after the third and 6th day of the feeding of dsRNA treated diet. The change in weight was calculated by subtracting the initial weight (IW) from the final weight (FW) as:

WG = FW-IW

2.5. Validation of dsRNA effect through qRT-PCR

Using qRT-PCR, the level of expression for each gene transcript was validated. The primer-BLAST tool in NCBI (https://www.ncbi.nlm.nih.gov/tools/primer-blast/) was used to design PCR primers for targeted genes. Total RNA was extracted from a set of test larvae (3 larvae

pooled in each set) as described above after seven days of treatment. First-strand cDNA was generated from 1 μg of RNA using oligo dT primers and MMLV reverse transcriptase (Thermofisher). The real-time PCR detection system (CFX-96 Touch™, BioRad, USA) was used to perform qRT-PCR assays. The qRT-PCR reactions were prepared using 50 ng cDNA, 0.5 μM of each gene specific primers, 10 μl of SYBR Green Master Mix (BioRad, USA) and nuclease-free water to a final volume of 20 μl. The universally expressed tubulin gene of RPW was used as a housekeeping gene to normalize the expression levels of individual genes in the experiment. The relative expression of the targeted genes was calculated following Livak and Schmittgen [36]. All qRT-PCR assay was repeated twice with three biological replicates.

4.7. Statistical analysis

The data were analyzed using a one-way ANOVA test with a significance level of 0.05 ($P \leq 0.05$). Tukey's multiple comparison test was then used to find the difference between treatments ($P \leq 0.05$).

3. Results

3.1. Effect of dsRNAs on mortality of larval Stages of RPW

The effects of feeding two larval instars of the RPW with dsRNAs targeting ECR, SCP, actin, and CBP were investigated. Table 2 shows the percent mortality of the third instar RPW larvae fed on four different dsRNAs. There was a significant difference in mortality rates between the four groups ($P \leq 0.05$). As dsRNA works slowly, data were recorded 3 and 6 days after treatment (DAT). At 3 DAT, higher mortality rate (53.19±3.86%) was observed in the larvae fed on actin-dsRNA. The mortality from ECR and CBP-dsRNA was the lowest (37.81 ± 6.05%) among the

treatments. At 6 DAT, the mortality of third instar larvae fed on different dsRNAs was increased in parallel to the increasing time of feeding. The mortality rate was higher, i.e., 86.05±5.58%, in actin-fed third instar RPW larvae. While the lowest mortality was found in for ECR, viz. 65.00±4.87%. No mortality was observed in the control treatments at 3 and 6 DAT ($P \leq 0.05$).

Similarly, in the fifth instar larvae, a significant difference in mortalities was observed caused by the four tested genes ($P \leq 0.05$). The larvae fed on actin-dsRNA showed the highest mortality (49.33±4.73%), while the lowest mortality (33.95±3.80%) was observed due to ECR-dsRNA at 3 DAT (Table 2). Meanwhile, the mortality rate of the fifth instar RPW larvae was highest, i.e., 65.12±7.85% in actin-fed fifth instar RPW larvae and the lowest mortality was found in ECR, viz. 49.33±4.73% at 6 DAT. Comparatively, in the control treatments, no mortality was observed at 3 and 6 DAT ($P \leq 0.05$).

3.2. Effects of dsRNAs on Survival of Two Larval Stages of RPW

The survival rate of 3rd instar larvae fed on dsRNA-treated diet differed significantly between the five treatments ($P \leq 0.05$). After 5 days, the control treatment has a significantly higher survival rate (100%) than the other treatments. While significantly lower survival rates were observed in RPW larvae fed on SCP (60%) and actin (68%), respectively. The survival rate of CBP and EcR ranged between 84-88%. After 10 days of incubation, the survival rates of third instar larvae treated with SCP and actin dsRNAs decreased to 40% and 48%, respectively. While the survival rate of CBP was 64% and EcR was 68%. Whereas a 100% survival rate was observed in the control treatment. After 15 days of incubation, the survival rate in both treatments (SCP and actin dsRNAs) was significantly decreased to 28%. At the end of the experiment, after 20 days of incubation, the survival rate was reduced to a significantly lowest value, i.e. 24% and 28% in

SCP and actin treatments, respectively. While the control larvae depicted the highest survival rate, viz. 96% after 20 days of treatment (Figure 2 A).

The survival rates of the 5th instar larvae fed on dsRNA treated diet showed no significant differences after five days of incubation with 84%, 84%, 92%, 96% and 100% survival rates in SCP, actin, CBP, ECR and control, respectively ($P > 0.05$). However, after 10 days of incubation, relatively lower survival rates were found in actin (64%) and SCP (68%), fol-lowed by CBP (72%) and EcR (76%), respectively, compared to the control (100%). After 10 days of incubation, the survival rates of the 5th instar larvae in the actin, SCP, CBP, and EcR groups were 44%, 48%, 52%, and 56%, respectively. After 20 days of incubation, the survival rates in these groups were 36%, 44%, 40%, and 48%, respectively. Meanwhile, the control larvae depicted the highest survival rate, viz. 96% after 20 days of incubation (Figure 2B).

3.3. Weight Gain in Different Stages of RPW Due to dsRNA Feeding

Data regarding weight changes depicted significant differences between the treatments. After 3 and 6 days of exposure, the 3rd and 5th instar larvae showed significant weight changes ($P \leq 0.05$). The initial weight of the third instar larvae ranged from 0.93 to 1.01 g. The control group had a significantly higher final weight (2.83 ± 0.05 g) than the dsR-NA-treated groups, which had significantly lower final weights (1.53–1.88 g) after 3 days of treatment. After 6 days of treatment, the larval weights of the CBP and ECR treatment groups were not significantly different from the control (2.90-3.15 g). However, the larval weights of the SCP and actin treatment groups were significantly lower (2.30-2.36 g) (Figure 3A).

The initial weight of the fifth instar larvae varied from 3.87 g to 4.15 g in all treatments. After 3 days, no significant difference was observed in the weight gain of control (4.79±0.01 g) and ECR

treatment (4.73±0.01 g). Whereas significantly lower weights were observed in the larvae treated with SCP, actin and CBP dsRNAs, ranging between 4.30-4.49 g (Figure 3B). After 6 days of treatment, the larval weight was significantly re-duced only in SCP and actin-treated larvae ranging between 4.64-4.78 g compared to other treatments, including control (5.24-5.65 g) (Figure 3A).

3.4. Effect of dsRNAs on Developmental time of Different Larval Instars of RPW

The effects of dsRNAs on larval development were measured in third instar larvae. All treatment groups showed significant differences in larval development time ($P \leq 0.05$) (Table 3). In the 3rd, 4th, 7th, 8th, 9th and 10th larval instars, all treatments had significantly shorter durations than the control. However, there were no significant differences in duration between the dsRNA treatments (Table 3). The duration in the SCP and actin treatments was the lowest in the aforementioned larval instars. The duration of the fifth instar was significantly lower in ECR, SCP and actin treatments compared to CBP and control. In the sixth instar, SCP significantly lowered the duration period (4.60±0.24 days) than the other three treatments (5.80-4.80 days). Finally, at the 11th instar, the duration was significantly reduced in the ECR, SCP and Actin treatments compared to CBP and control (Table 3).

Similarly, all treatment groups showed significant differences in development time of 5th instar larvae ($P \leq 0.05$). In the 5th, 6th, 7th and all other larval instars, all treatments had significantly shorter durations than the control. The duration in the SCP and actin treatments was the lowest in the aforementioned larval instars. The duration of the 5th instar was lower in SCP (3.80±0.20 days) and actin (4.20±0.37 days) treatments as compared to the control (6.80±0.37 days). In the 6th instar, SCP and actin significantly lowered the duration period (4.00±0.32 days) than the control

(7.00±0.45 days). However, at the 11th instar, there was no significant difference in the duration (Table 4).

3.5. Effect of dsRNAs on pupal duration of RPW

Pupal duration recorded in the treated 3rd instar larvae after their exposure to dsRNA for 3 days varied between the treatments ($P \leq 0.05$). Pupal duration recorded was lower in SCP and actin treatment were 12.20±0.20 and 12.00±0.32 days, while it was significantly higher in the control (14.00±0.32). Similarly, lower pupal duration was observed in actin (11.20±0.24 days) and SCP (11.40±0.20 days). While, the pupal duration in the control was significantly higher viz., 13.80±0.20 days (Figure 4).

3.6. *Effect of dsRNAs on adult emergence*

Adult emergence from the pupae of treated 3rd instar larvae after their exposure to dsRNA for 3 days varied between the treatments ($P \leq 0.05$). Adult emergence was lower in SCP and actin treatment i.e., 8.00±4.89%, while it was significantly higher in the control (84.00±4.00%). Similarly, lower adult emergence from pupae of actin (20.00±4.89%) and SCP (24.40±4.00%) treated 5th instar larvae was observed. While, the adult emergence in the control was significantly higher viz., 88.00±4.89% (Figure 4).

3.7. Relative expression in RPW larvae

A significant reduction was observed in the expression of all studied gene transcripts in the third instar larvae fed on the respective dsRNA-treated diet compared to controls (untreated) (Figure 4A). The control group was measured separately for each gene using their individual primers (Table 1). The expression of the control group was taken as 1.0000 in all cases. Relative gene

expression in the case of different dsRNA treatments varied significantly between the treatments. The relative expression of ECR, SCP, actin, and CBP were 0.1 (90% decrease), 0.04 (96% decrease), 0.03 (97% decrease), and 0.09 (91% decrease), respectively It was found that the transcript level of SCP and actin genes was most significantly reduced compared to other treatments in the third instar larvae.

4. Discussion

The global spread of RPW has made it an economically important pest in date palm production. Many local or regional management practices and chemical-based pesticides, are routinely used to control RPW. However, the injudicious use of chemical insecticides has instigated specific serious problems, including insect resistance development, human health hazards, and environmental threats [13,37]. Our study showed that among four different dsRNAs treatments, the mortality rate of the third and fifth instar RPW larvae fed on actin-dsRNAs was significantly highest at 3 and 6 DAT. While, the survival rate of third and fifth instar larvae fed on SCP and Actin-dsRNAs was significantly reduced among all treatments and compared to the control after 20 days of incubation. Our results corroborate the previous studies where a significantly lower survival rate was reported in beetles due to the downregulation of SCP transcripts [38,39]. When SCP levels are low, the insect's development is compromised. This is because SCPs, such as trypsins and chymotrypsins, are the main digestive enzymes in the insect's midgut. These enzymes break down proteins into amino acids, which are essential for growth and development [25,40,41]. Previous studies have shown a direct relationship between insect survival and actin expression [27–29,42]. The actin gene is well-documented for its role in many cellular processes, including muscle contraction, cell motility, cytoskeletal structure, cell division, intracellular transport, and cell differentiation. The three main isotypes of actin (alpha, beta, and gamma) each

play a specific role in these processes. For example, alpha-actin is essential for muscle contraction, while beta-actin is involved in cell motility. Gamma-actin is involved in cytoskeletal structure and cell division [29,43].

Weight gain is an important indicator of growth and development in chewing in-sects. It can also be used to assess the efficacy of ingested dsRNA [9]. In the current study, we incubated RPW larvae and analyzed their weight gain before and after the experiment. The larvae that were fed actin and SCP-dsRNAs gained significantly less weight than the control group. These findings are similar to previous research that has shown that down-regulation of actin can lead to decreased insect development [44]. In fact, weight is directly linked with actin, as in the myofibrils of muscle cells. Where actin serves as the primary contractile component and ATP synthase is the enzyme responsible for generating cellular energy [45,46].

The developmental time of RPW is an important factor in assessing the population growth of date palm plants. Larvae fed on dsRNA developed faster than control larvae, taking 3.80-5.40 days to reach each stage, compared to more than 6 days for control larvae. This reduction in larval developmental time has been reported previously [47–49]. Rewitz and Gilbert [49] found that the CBP protein mediates the growth hormones in insects, and that any interference in this enzyme may lead to a change in larval development. Similarly, the SCP protein is associated with cuticle differentiation in Drosophila melanogaster [50]. Qiao, Xiong, Wang, He, Chen, Tong, Hu, Li, Gai and Xin [48] found that changes in cuticle protein can affect larval growth in silkworms. Whereas, EcR and actin signaling influence neuronal remodeling by regulating the expression of the 20E receptor. This signaling pathway may also play a role in larval development.

To assess the efficiency of dsRNAs in silencing targeted genes, we analyzed the transcript levels of these genes in RPW larvae. qRT-PCR results showed that the transcript levels of all targeted genes were significantly reduced in third and fifth instar larvae. The most efficient silencing was observed for the actin gene, followed by SCP, CPB, and EcR (Figure 3). The reduction in transcript levels of targeted genes established the efficacy of dsRNAs. The results are in agreement with the earlier studies [34,47].

The present study showed that RPW larvae feeding on different dsRNAs exhibited a reduction in transcript levels, lower body weight gain and pronounced metamorphosis effects due to the sub-lethal effects of dsRNAs. Among these dsRNAs, actin dsRNAs showed more efficient, lethal and sub-lethal effects on insect survival when compared to other genes. These results are encouraging for controlling the notorious RPW pest. The study used an alternative, effective, and reliable method of developing small dsRNA molecules in the form of small bio-preparations. This could lead to a better integrated pest management approach.

5. Conclusions

Red palm weevil threatens date palm production worldwide, especially in Saudi Arabia. An adequate eco-friendly management approach can help better circumvent this notorious pest. Silencing four RPW growth-related genes produced promising results in this study. The results improvise essential evidence that the survival rate, larval weight and development time of third and fifth instar larvae were significantly affected by silencing the actin and SCP genes. Future studies should focus on practicing and optimizing an appropriate delivery method for dsRNAs in insect larvae, viz. nanoparticles or more efficient bacterially expressed systems. The results

further support the adoption of RNAi as an alternate technology to the conventional RPW controlling strategies.

Supplementary Materials: Not applicable

Author Contributions: Conceptualization, J.M.A., A.A.R. and M.N.S.; methodology, M.N.N. and K.M.; software, A.B. and M.N.N.; validation, M.N.S. and M.N.N.; formal analysis, A.B.. and K.M.; investigation, M.N.S., M.N.N. and K.M..; funding acquisition, J.M.A., A.A.R., M.N.S. and. H.E.; resources, J.M.A. and M.N.N.; data curation, M.N.S. and A.A.R.. The first draft of the manuscript was written by M.N.S. and M.N.N. The final draft reviewing and editing was done by A.B. and K.M. All authors have read and agreed to the final version of the manuscript.

Funding: This project was financially supported King Abdulaziz City for Science and Technology (KACST), Saudi Arabia through the Project No. 11BIO1803-06

Institutional Review Board Statement: NA

Informed Consent Statement: NA

Data Availability Statement: All the data can be accessed under accession number ON756218-33 and ON843661-64 in the NCBI GenBank database (https://www.ncbi.nlm.nih.gov/nucleotide/).

Acknowledgments: In this section, you can acknowledge any support given which is not covered by the author contribution or funding sections. This may include administrative and technical support, or donations in kind (e.g., materials used for experiments).

Conflicts of Interest: The authors declare no conflict of interests.

References

1. Dhehibi B, Salah MB, Frija A. Date palm value chain analysis and marketing opportunities for the Gulf Cooperation Council (GCC) countries. In Agricultural Economics-Current Issues; IntechOpen; 2018.

2. Al-Khayri JM. et al. Genetic and morphological diversity assessment of five kalanchoe genotypes by SCoT, ISSR and RAPD-PCR markers. Plants. 2022;11: 1722.

3. FAO. Food and Agriculture Organization of the United Nations; 2021.

4. Olivier V, Monsempes C, François MC, Poivet E, Jacquin- Joly E. Candidate chemosensory ionotropic receptors in a Lepidoptera. Insect Mol Biol. 2011;20: 189–199.

5. Manee MM, Alqahtani FH, Al-Shomrani BM, El-Shafie HA, Dias GB. Omics in the red palm weevil *Rhynchophorus ferrugineus* (Olivier) (Coleoptera: Curculionidae): A bridge to the pest. Insects. 2023;14: 255.

6. Belvedere S, Arnone S, Cristofaro M, La Marca A, De Biase A. Paternity analyses for the planning of SIT projects against the red palm weevil. Insects. 2023;14: 326.

7. Mahadi NA. et al. Comparative Study of Red Palm Weevil (RPW), *Rhynchophorus ferrugineus* (Coleoptera: Dryophthoridae) Reproduction Reared Using Different Diets. Am J Entomol. 2022;6: 110–115.

8. Abdel-Baky N. et al. Monitoring of infestation percentages of the invasive red palm weevil, *Rhynchophorus ferrugineus* (Coleoptera: Curculionidae), and management tactics: a six-year study. Braz J Biol. 2022;82.

9. Nurashikin-Khairuddin W. et al. A review of entomopathogenic nematodes as a biological control agent for red palm weevil, *Rhynchophorus ferrugineus* (Coleoptera: Curculionidae). Insects. 2022;13: 245.

10. Muhammad A. et al. Spätzle homolog-mediated Toll-like pathway regulates innate immune responses to maintain the homeostasis of gut microbiota in the red palm weevil, *Rhynchophorus ferrugineus* Olivier (Coleoptera: Dryophthoridae). Front Microbiol. 2020;11: 846.

11. Wakil W. et al. Resistance to commonly used insecticides and phosphine fumigant in red palm weevil, *Rhynchophorus ferrugineus* (Olivier) in Pakistan. PloS One. 2018;13: e0192628.

12. Habood EA, Sayed R, Rizk S, El-Sharkawy AZ, Badr NF. Nanochitosan to control the red palm weevil *Rhynchophorus ferrugineus* (Olivier) (Coleoptera: Curculionidae). J Rad Res Appl Sci. 2022;15: 7–17.

13. Budzinski H, Couderchet M. Environmental and human health issues related to pesticides: from usage and environmental fate to impact. Environ Sci Poll Res. 2018;25: 14277–14279.

14. Tan H, et al. Occurrence, distribution, and driving factors of current-use pesticides in commonly cultivated crops and their potential risks to non-target organisms: A case study in Hainan, China. Sci Total Environ. 2023;854: 158640.

15. Wu SF, et al. The evolution of insecticide resistance in the brown planthopper (*Nilaparvata lugens* Stål) of China in the period 2012–2016. Sci Rep. 2018;8: 1–11.

16. Bilal M, Freed S, Ashraf MZ, Rehan A. Resistance and detoxification enzyme activities to bifenthrin in *Oxycarenus hyalinipennis* (Hemiptera: Lygaeidae). Crop Prot. 2018;111: 17–22.

17. Kinareikina A, Silivanova E. Impact of insecticides at sublethal concentrations on the enzyme activities in adult *Musca domestica* L. Toxics. 2023;11: 47.

18. Cai Q, He B, Kogel KH, Jin H. Cross-kingdom RNA trafficking and environmental RNAi—nature's blueprint for modern crop protection strategies. Curr Opin Microbiol. 2018;46: 58-64.

19. Laudani F. et al. RNAi-mediated gene silencing in *Rhynchophorus ferrugineus* (Oliver) (Coleoptera: Curculionidae). Open Life Sci. 2017;12: 214–222.

20. Rasool, K.G. et al. Silencing of vitellogenin gene contributes to the promise of controlling red palm weevil, *Rhynchophorus ferrugineus* (Olivier). Sci Rep. 2021;11: 21695.

21. Khalil S, El-Gamal S, Ibrahim S, Elateek, S. Characterization, expression analysis and RNAi-mediated knockdown of two aquaporin genes in the cotton leafworm, *Spodoptera littoralis* (Lepidoptera: Noctuidae). Eur J Entomol. 2023;120: 151–125.

22. Lu JB. et al. RNAi- mediated silencing of an egg- specific gene *Nllet1* results in hatch failure in the brown planthopper. Pest Manag Sci. 2023;79: 415–427.

23. Zhang Q. et al. Pannier is a key regulator of embryogenesis, pupal development and female reproduction in the insect pest *Bactrocera dorsalis*. Pest Manag Sci. 2023;79: 1352–1361.

24. Feng Y. et al. In vitro binding effects of the ecdysone receptor− binding domain and PonA in *Plutella xylostella*. Molecules. 2023;28: 1426.

25. Huang X. et al. The new effector AbSCP1 of foliar nematode (*Aphelenchoides besseyi*) is required for parasitism rice. J Integr Agr. 2022;21: 1084–1093.

26. Huang X, Xu CL, Chen WZ, Chen C, Xie H. Cloning and characterization of the first serine carboxypeptidase from a plant parasitic nematode, *Radopholus similis*. Sci Rep. 2017;**7**: 4815.

27. Wu M. et al. Efficient control of western flower thrips by plastid-mediated RNA interference. Proc Nat Acad Sci. 2022;119: e2120081119.

28. Kim M, Robich RM, Rinehart JP, Denlinger DL. Upregulation of two actin genes and redistribution of actin during diapause and cold stress in the northern house mosquito, *Culex pipiens*. J Insect Physiol. 2006;52: 1226–1233.

29. He W. et al. Positional effects of double-stranded RNAs targeting β-Actin gene affect RNA interference efficiency in Colorado potato beetle. Pestic Biochem Physiol. 2022;184: 105121.

30. Tetreau G. et al. Analysis of chitin-binding proteins from *Manduca sexta* provides new insights into evolution of peritrophin A-type chitin-binding domains in insects. Insect Biochem Mol Biol. 2015;**62**: 127–141.

31. Rehman KU. et al. Insect-Derived chitin and chitosan: A still unexploited resource for the edible insect sector. Sustainability. 2023;15: 4864.

32. Aldawood AS et al. Semi-artificial diet developed for the successful rearing of red palm weevil: *Rhynchophorus ferrugineus* (Coleoptera: Dryophthoridae) in the laboratory. J King Saud Univ Sci. 2022;34: 102272.

33. Kamath RS, Ahringer J. Genome-wide RNAi screening in Caenorhabditis elegans. Methods 2003;30: 313–321.

34. Zhu F, Xu J, Palli R, Ferguson J, Palli SR. Ingested RNA interference for managing the populations of the Colorado potato beetle, *Leptinotarsa decemlineata*. Pest Manag Sci. 2011;67: 175–182.

35. Al- Ayedh H, Rizwan- ul- Haq M, Hussain A, Aljabr AM. Insecticidal potency of RNAi-based catalase knockdown in *Rhynchophorus ferrugineus* (Oliver) (Coleoptera: Curculionidae). Pest Manag Sci. 2016;72: 2118–2127.

36. Livak KJ, Schmittgen TD. Analysis of relative gene expression data using real-time quantitative PCR and the 2− ΔΔCT method. Methods, 2001;25: 402-408.

37. Tiwari S, Pelz-Stelinski K, Mann RS, Stelinski LL. Glutathione transferase and cytochrome P450 (general oxidase) activity levels in *Candidatus Liberibacter* asiaticus-infected and uninfected Asian citrus psyllid (Hemiptera: Psyllidae). Ann Entomol Soc Am. 2011;104: 297–305.

38. Meng FL. et al. RNA interference mediated serine protease gene (Spbtry1) knockdown affects growth and mortality in the soybean pod borer (Lepidoptera: Olethreutidae). Fla. Entomol. 2017;100: 607–615.

39. Zha W. et al. Knockdown of midgut genes by dsRNA-transgenic plant-mediated RNA interference in the hemipteran insect *Nilaparvata lugens*. PloS One. 2011;6: e20504.

40. Gatehouse JA. Prospects for using proteinase inhibitors to protect transgenic plants against attack by herbivorous insects. Curr. Protein Pept. Sci. 2011;12: 409–416.

41. Marshall S. et al. Serine proteases identified from a *Costelytra zealandica* (White) (Coleoptera: Scarabaeidae) midgut EST library and their expression through insect development. Insect Mol Biol. 2008;17: 247–259.

42. Bar L, Czosnek H, Sobol I, Ghanim M, Hariton Shalev A. Downregulation of dystrophin expression in pupae of the whitefly *Bemisia tabaci* inhibits the emergence of adults. Insect Mol Biol. 2019;28: 662–675.

43. Galetto L. et al. The major antigenic membrane protein of "*Candidatus phytoplasma* asteris" selectively interacts with ATP synthase and actin of leafhopper vectors. PloS One. 2011;6: e22571.

44. Rosa C, Kamita SG, Falk BW. RNA interference is induced in the glassy winged sharpshooter *Homalodisca vitripennis* by actin dsRNA. Pest Manag Sci. 2012;68: 995–1002.

45. Upadhyay SK. et al. RNA interference for the control of whiteflies (*Bemisia tabaci*) by oral route. J Biosci. 2011;36: 153–161.

46. Wuriyanghan H, Rosa C, Falk BW. Oral delivery of double-stranded RNAs and siRNAs induces RNAi effects in the potato/tomato psyllid, *Bactericerca cockerelli*. PloS One. 2011;6: e27736.

47. Naqqash MN, Gökçe A, Aksoy E, Bakhsh A. Downregulation of imidacloprid resistant genes alters the biological parameters in Colorado potato beetle, *Leptinotarsa decemlineata* Say (chrysomelidae: Coleoptera). Chemosphere. 2020;240: 124857.

48. Qiao L. et al. Mutation of a cuticular protein, BmorCPR2, alters larval body shape and adaptability in silkworm, *Bombyx mori*. Genetics. 2014;196: 1103–1115.

49. Rewitz KF, Gilbert LI. Daphnia Halloween genes that encode cytochrome P450s mediating the synthesis of the arthropod molting hormone: evolutionary implications. BMC Evol Biol. 2008;8: 1–8.

50. Kenney E. et al. A putative lysozyme and serine carboxypeptidase from *Heterorhabditis bacteriophora* show differential virulence capacities in *Drosophila melanogaster*. Dev Comp Immunol. 2021;114: 103820.

Figures Click here to access/download;Figure;Figures.docx

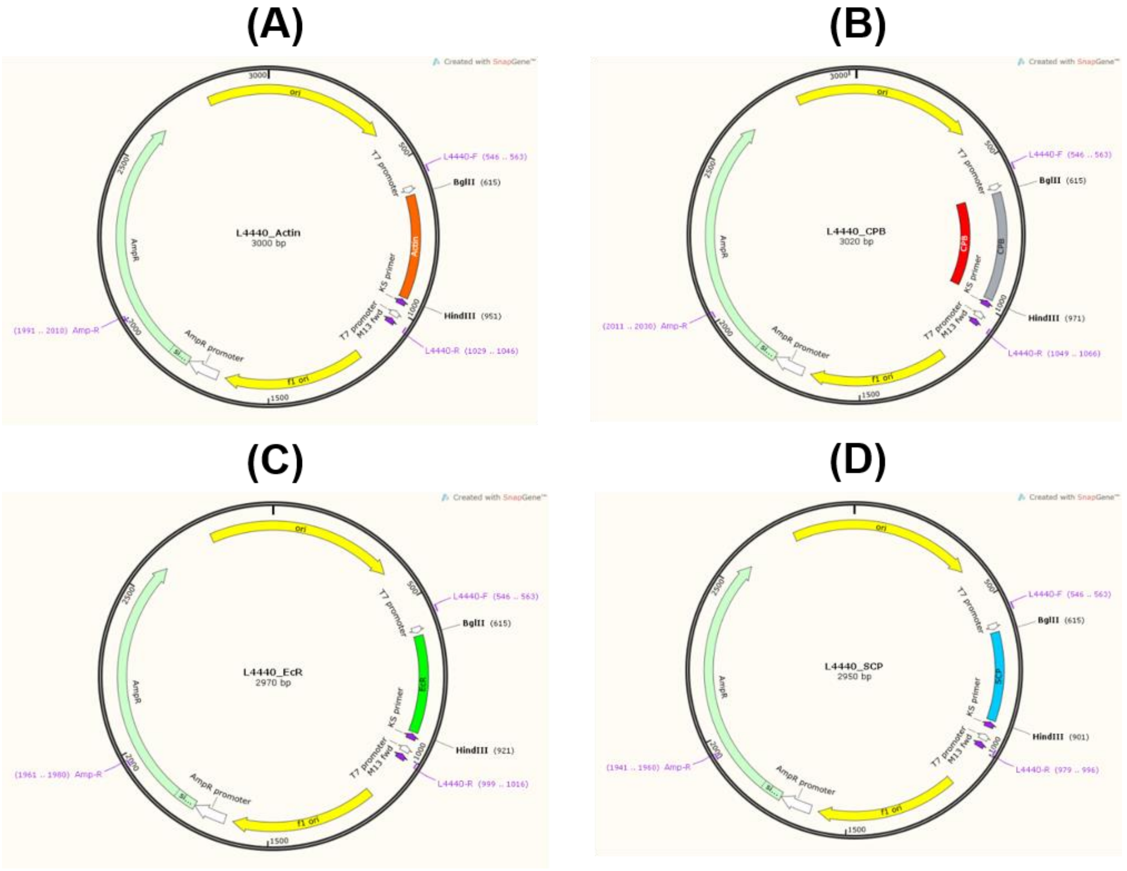

Figure 1: The fragments of actin **(A)**, CPB **(B)**, EcR **(C)** and SCP **(D)** genes of RPW were cloned in L4440 vector as were named as L4440_Actin, L4440_CPB, L4440_EcR and L4440_SCP. The maps were developed using SnapGENE.

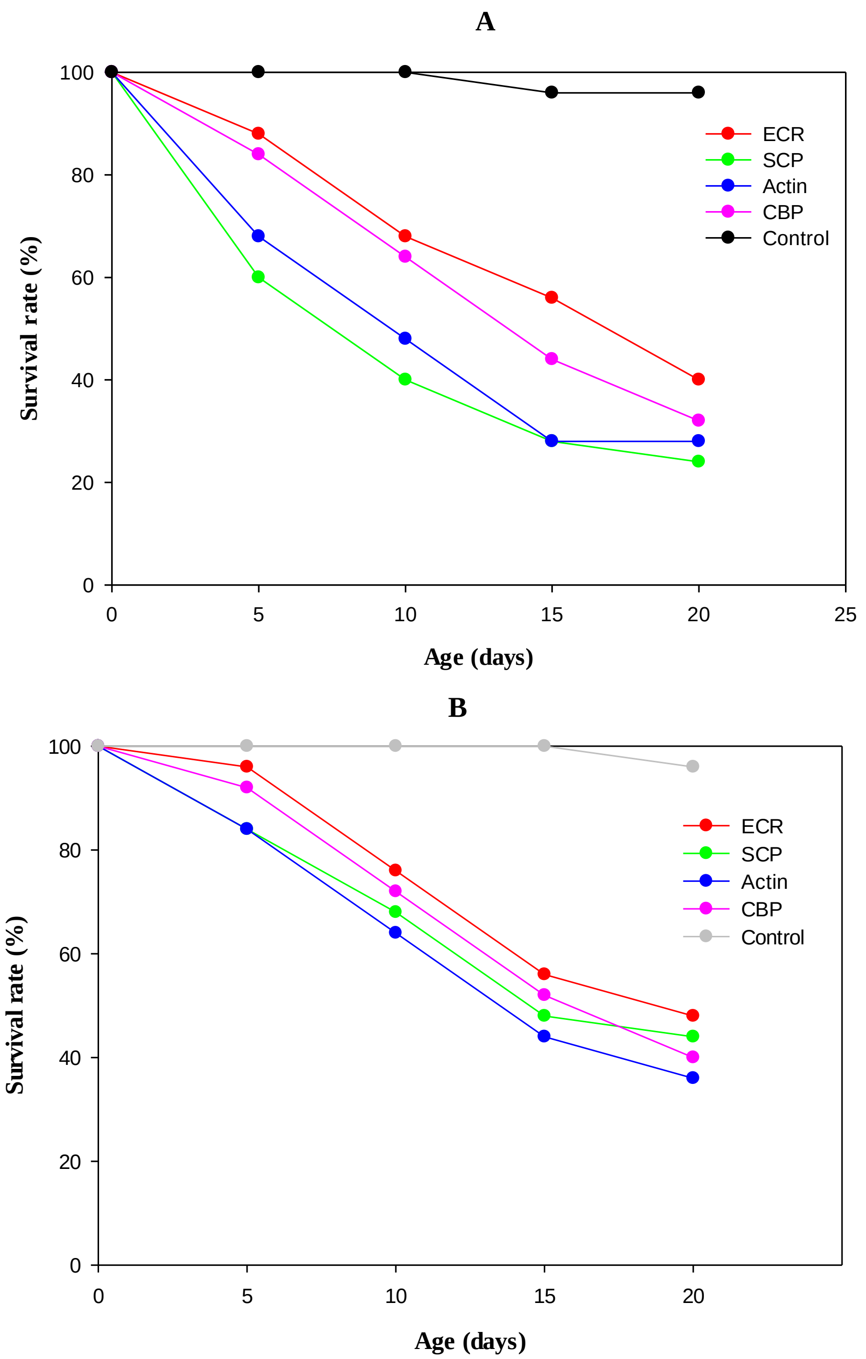

Figure 2: Survival of **(A)** third instar and **(B)** fifth instar of RPW after feeding on different dsRNA**s**

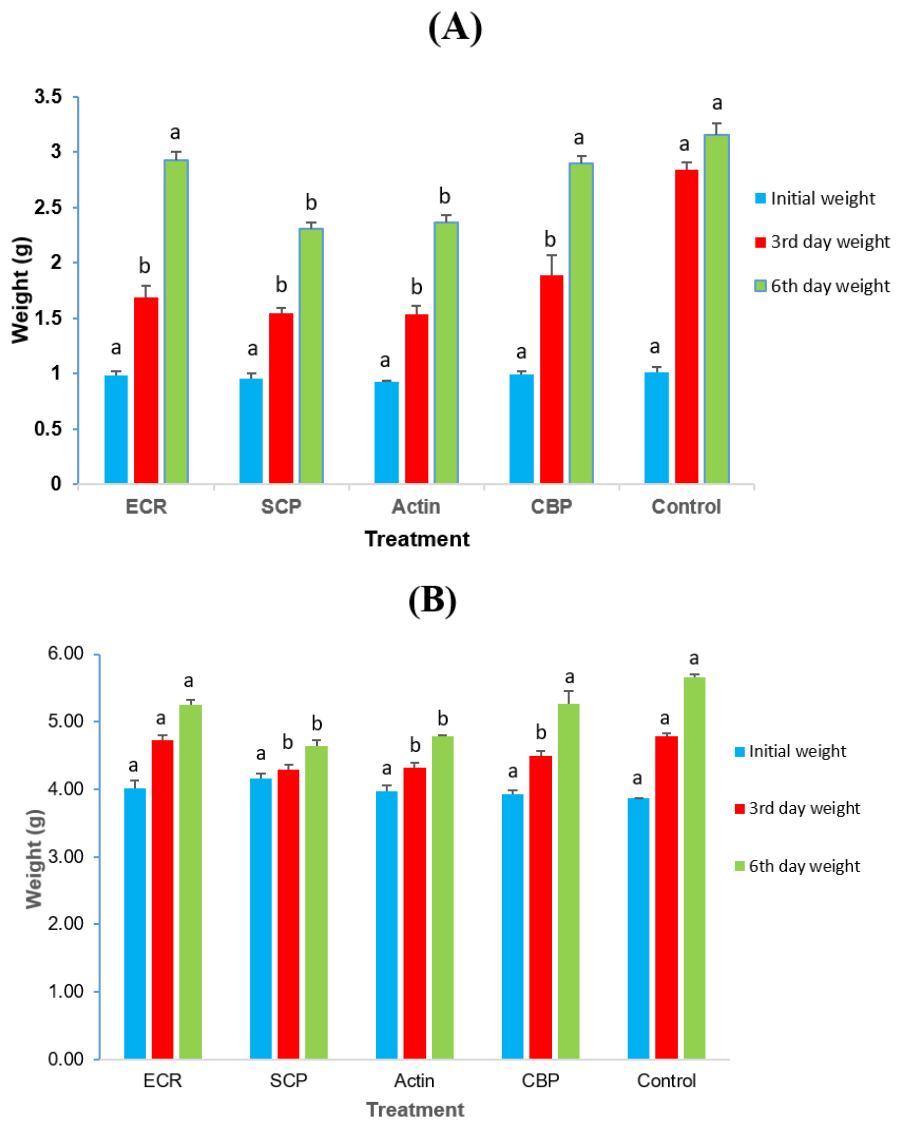

Figure 3: Weight change in **(A)** third instar and **(B)** fifth instar of RPW after feeding on different dsRNAs

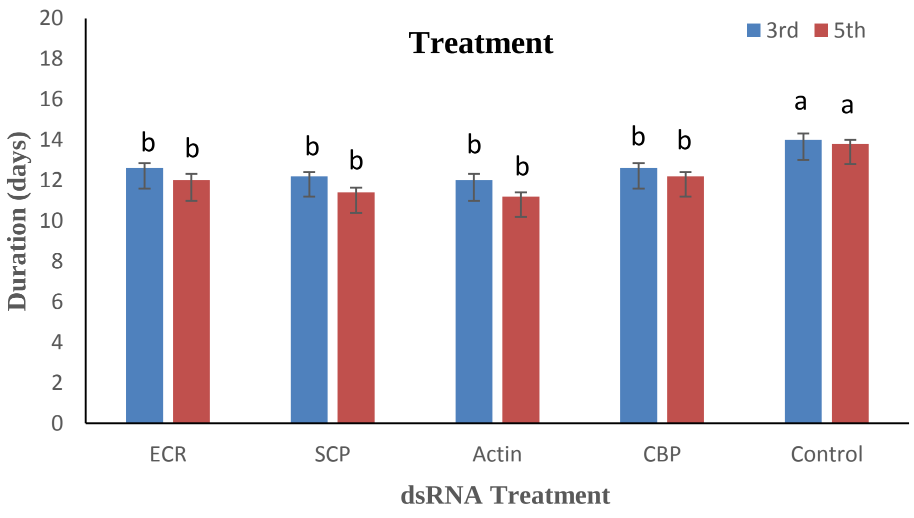

Figure 4: Pupal duration of (A) 3rd instar and (B) **5**th instar larvae of RPW after feeding on different dsRNA**s**

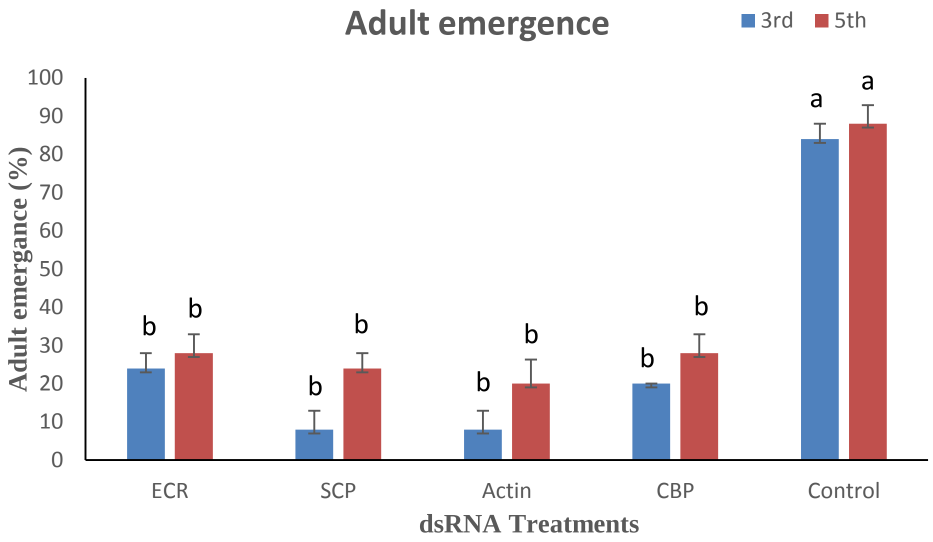

Figure 5: Adult emergence of 3rd instar and **5**th instar of RPW after feeding on different dsRNA**s**
