# Supplementary material for: Sprayable RNAi for silencing of important genes to manage red palm weevil, Rhynchophorus ferrugineus (Coleoptera: Curculionidae)
Source: PLoS One. 2024 Oct 24;19(10):e0308613. doi: 10.1371/journal.pone.0308613 (PMC11501036; doi:10.1371/journal.pone.0308613)
Supplement: Attachment — Submitted filename: Response to reviewers.docx [file pone.0308613.s003.docx]

We are grateful to the reviewers for their thorough evaluation of our manuscript titled "Sprayable RNAi for silencing of important genes to manage red palm weevil, *Rhynchophorus ferrugineus* (Coleoptera: Curculionidae)." We have carefully considered each comment and have made significant revisions to address the reviewers' concerns. Below, we provide a detailed response to each point raised by the reviewers, indicating how we have modified the manuscript accordingly.

Reviewer #1:

1. Page 20: Sentence Clarity

- Comment: "Pupal duration recorded was lower in SCP and actin treatment were 12.20±0.20 and 12.00±0.32 days, while it was significantly higher in the control (14.00±0.32)."

- Response: We have rewritten this sentence for clarity. It now reads: " The pupal duration was lower in the SCP and actin treatments (12.20±0.20 and 12.00±0.32 days, respectively), whereas it was significantly higher in the control (14.00±0.32)."

2. Page 23: Clarifications Needed

- Comment: Define “reduction in transcript levels,” “pronounced metamorphosis effects,” and “efficient effect.”

- Response: We have clarified these terms. “Reduction in transcript levels” now specifies “expression levels of the targeted gene.” “Pronounced metamorphosis effects” are defined by observable differences in developmental stages, particularly changes in mortality rates and developmental timing. Terms are revised as “body weight gain, and different preadult length, weight, and survival rates.”

3. Figure 1: Labeling Issue

- Comment: "vector as were named as" should be "vector and were designated as"

- Response: This correction has been made in the manuscript.

4. Figures 3-5: Clarification of Letters

- Comment: Explanation needed for letters 'a' and 'b.'

- Response: We have added a legend explaining that the letters 'a' and 'b' indicate statistically significant differences between groups, with the same letter denoting no significant difference.

5. Figure 4: Clarification of (A) and (B)**

- Comment: Clarify what (A) and (B) indicate.

- Response: The legend now specifies that (A) and (B) represent different experimental conditions or treatments.

6. Table 1: Missing Table

- Comment: Table 1 is missing.

- Response: We apologize for this oversight. Table 1 has been included in the revised manuscript.

Reviewer #2:

We thank Reviewer #2 for their positive feedback and constructive suggestions. We have incorporated their specific comments from the attached manuscript to improve clarity and depth.

Reviewer #3:

1. Selection of 3^rd^ and 5^th^ Instar

- Comment: Justification needed for choosing 3rd and 5th instar.

- Response: We have added a detailed explanation in the Materials and Methods section, justifying the selection based on previous studies showing these stages are critical for assessing RNAi efficacy due to their active feeding and development phases.

2. Depth of Experimental Content

- Comment: Limited experimental content.

- Response: Modified methodology of earlier studies conducted by Naqqash et al. (2020) was used for the experiments (Naqqash, M.N., Gökçe, A., Aksoy, E. and Bakhsh, A., 2020. Downregulation of imidacloprid resistant genes alters the biological parameters in Colorado potato beetle, Leptinotarsa decemlineata Say (chrysomelidae: Coleoptera). *Chemosphere*, *240*, p.124857).

Reviewer #4:

1. Details on Survival Analysis

- Comment: Prefer Kaplan-Meier analysis for survival data.

- Response: We have conducted a Kaplan-Meier survival analysis and included these results in the revised manuscript.

2. Figure 3: Y-axis and Scale Harmonization

- Comment: Harmonize Y-axis and scale.

- Response: The Y-axis and scale in Figure 3 have been harmonized for consistency. However, for follow up time we have followed already available literature which has suggested follow up time of 3-6 days.

4. Effect of Selected Genes

- Comment: Clarify the direct effects of selected genes on weight gain.

- Response: We have discussed the roles of the targeted genes in physiological and morphological traits, explaining their indirect effects on weight gain.

5. Figure for Pupal Duration and Gene Expression

- Comment: Revision of figures and inclusion of gene expression data.

- Response: We have revised the figures for clarity and included a new figure illustrating the relative expression levels of the targeted genes. Statistical analyses for these data have also been added.

We believe that these revisions have significantly improved the manuscript. We are grateful for the reviewers' insights, which have helped us enhance the quality and clarity of our work. We look forward to the possibility of our revised manuscript being considered for publication.
